# A New Paradigm of Bispecific Antibodies in Clinical Management of Gastrointestinal Cancers

**DOI:** 10.32604/or.2026.078825

**Published:** 2026-06-16

**Authors:** Huimin Zhao, Xiuran Wang, Zhimeng Fan, Ai Sun, Changhua Zhang, Chunhui Sun

**Affiliations:** 1 Digestive Diseases Center, Research Center, The Seventh Affiliated Hospital of Sun Yat-Sen University, Shenzhen, China; 2 College of Laboratory Animal Science, Shandong First Medical University, Jinan, China; 3 Division of Oncology, Department of Clinical Sciences, Lund University, Lund, Sweden; 4 Breeding and Biotechnology Research Laboratory, Beijing Academy of Agriculture and Forestry Sciences, Beijing, China

**Keywords:** Gastrointestinal cancers, bispecific antibodies, molecular targets, tumor-associated antigen, immunotherapy

## Abstract

Gastrointestinal (GI) cancers represent a significant global health burden, characterized by high incidence, poor prognosis, and limited response to monotherapies. The advent of bispecific antibodies (BsAbs) has introduced a novel therapeutic paradigm, enabling dual targeting of tumor-associated antigens and immune effectors to enhance antitumor immunity. This review provides a comprehensive overview of recent advances in bsAb-based immunotherapy across major GI malignancies, including colorectal, gastric, pancreatic, biliary tract, esophageal, and liver cancers. We summarize key molecular targets and highlight representative clinical candidates such as CEA-TCB and RG7802. The discussion extends to innovative strategies involving BsAbs in modulating the immunosuppressive tumor microenvironment and overcoming resistance mechanisms. Furthermore, we explore promising combination therapies involving BsAbs with chemotherapy and immune checkpoint inhibitors (ICIs). The role of artificial intelligence in target prediction and structure optimization is also examined, alongside the potential of personalized immune strategies. Despite promising therapeutic potential, BsAbs face challenges including cytokine release syndrome (CRS), on-target/off-tumor toxicity, tumor heterogeneity-driven resistance, and tumor microenvironment (TME). Current strategies to improve therapeutic index encompass affinity-tuned designs, conditional activation mechanisms, biomarker-driven patient stratification, combination therapies, and artificial intelligence (AI)-guided optimization, warranting continued interdisciplinary research efforts. We conclude by outlining future research directions and emphasizing the importance of interdisciplinary, multicenter collaborations to accelerate clinical translation and maximize the therapeutic potential of BsAbs in GI oncology. In summary, the aim of this study is to critically evaluate the current landscape of BsAb-based immunotherapy in gastrointestinal cancers, synthesizing emerging molecular targets, evaluating clinical trial outcomes, identifying key challenges limiting therapeutic efficacy, and proposing evidence-based strategies to optimize future BsAb development.

## 1 Introduction

Gastrointestinal (GI) cancers represent a biologically and clinically heterogeneous group of malignancies, including gastric, colorectal, esophageal, pancreatic, and rare neuroendocrine tumors, remain a leading cause of global cancer mortality, with increasing incidence in younger populations [1,2,3,4]. Management of many gastrointestinal malignancies typically involves multimodal therapeutic strategies, integrating surgical resection, cytotoxic chemotherapy, radiotherapy, and targeted molecular interventions [3,4]. While disparities in diagnosis and care pathways, especially in low- and middle-income countries (LMICs) [5]. as well as complications such as treatment-induced immune responses and infections, underscore the urgent need for more precise and integrative therapies [6,7].

Despite the clinical success of monoclonal antibodies (mAbs) in various solid tumors [8,9,10], their therapeutic efficacy in GI malignancies remains suboptimal due to several intrinsic and extrinsic limitations, including tumor antigen heterogeneity, impaired tissue penetration, development of primary and acquired resistance, and immune evasion mechanisms [11,12,13]. Moreover, the effectiveness of mAbs such as trastuzumab and bevacizumab is often compromised by limited bioavailability, off-target toxicities, and stromal barriers within the TME [14,15].

**Figure 1:**
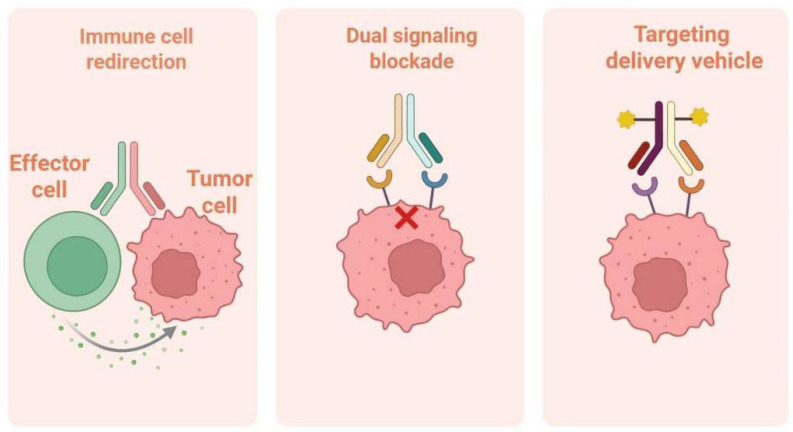
Schematic illustration depicting the principal mechanisms of action of bispecific antibodies in cancer immunotherapy. Core modalities include the recruitment of immune effector cells through simultaneous engagement with tumor targets, dual blockade of signaling pathways to suppress oncogenic drivers, and targeted delivery of therapeutic agents to enhance localized antitumor activity. Collectively, these strategies underscore the versatility and broad therapeutic potential of bispecific antibody platforms.

Bispecific antibodies (BsAbs) constitute a structurally and functionally heterogeneous class of engineered immunoglobulins capable of simultaneously binding two distinct antigens or epitopes [16,17]. BsAbs exert antitumor activity through multiple complementary mechanisms that collectively enhance immune surveillance and overcome tumor immune evasion (Fig. 1). One of the most extensively studied mechanisms is immune cell redirection. By simultaneously engaging a tumor-associated antigen (TAA) and a receptor on effector cells—such as CD3 on T cells or CD16 on natural killer (NK) cells—BsAbs physically bridge immune cells with malignant targets [18,19]. This proximity leads to direct cytotoxicity via perforin/granzyme release and activation of apoptosis pathways, independent of major histocompatibility complex (MHC) recognition [20,21]. The most well-known example is blinatumomab, a BiTE (bispecific T-cell engager) approved for acute lymphoblastic leukemia [22,23,24]. The success of blinatumomab in hematological malignancies provides valuable insights for solid tumor applications: it demonstrates that T-cell redirection can achieve potent antitumor activity even at low antigen densities, and that manageable toxicity profiles are achievable with appropriate step-up dosing strategies [20,25,26]. However, translating this success to GI cancers requires addressing unique challenges, including the immunosuppressive TME, physical barriers to T-cell infiltration, and heterogeneous antigen expression patterns characteristic of solid tumors [27,28,29].

Another key mechanism is dual signaling blockade, wherein BsAbs inhibit two distinct oncogenic or immunosuppressive pathways simultaneously [30,31]. For instance, BsAbs targeting both EGFR and HER2 [32], or VEGF and Ang-2 [33], have been shown to suppress redundant signaling cascades and angiogenesis more effectively than monospecific antibodies. Additionally, BsAbs can modulate the TME by concurrently neutralizing multiple immunosuppressive ligands or checkpoints [34,35]. Examples include BsAbs against PD-1/CTLA-4 [36,37] or PD-1/LAG-3 [38,39] are designed to synergistically enhance T-cell activation while mitigating adaptive resistance mechanisms commonly observed in immune checkpoint blockade therapy. Finally, BsAbs can serve as targeted delivery vehicles [40]. By binding a tumor antigen with one arm and conjugating toxins [41], cytokines [42], or radionuclides [43] to the other, BsAbs ensure precise delivery of potent agents directly to malignant cells. This approach maximizes efficacy while minimizing systemic exposure.

The rational development of BsAbs requires a systematic approach encompassing antibody screening, vector engineering, and the selection of an optimal expression system [44,45,46]. Antibody screening constitutes the first critical step and involves identifying parental monoclonal antibodies with high affinity, specificity, and non-overlapping epitopes suitable for dual targeting [44,45]. While hybridoma technology offers a stable source of monoclonals, its repertoire diversity is limited. In contrast, phage and yeast display libraries enable the selection of large antibody repertoires and allow affinity maturation under controlled conditions [47,48]. More recently, single B-cell cloning and next-generation sequencing of immune repertoires have facilitated the discovery of naturally occurring antibodies with reduced immunogenicity and improved biophysical properties [49,50]. Following antibody selection, vector design plays a decisive role in ensuring correct chain pairing and efficient expression. Bicistronic constructs using internal ribosome entry sites (IRES) or 2A self-cleaving peptides allow stoichiometric expression of two chains, although total yields may be reduced compared to dual-promoter plasmids, which offer independent transcriptional control at the expense of increased chain mispairing [51,52,53]. Codon optimization, signal peptide engineering, and Fc modification strategies such as “knobs-into-holes” or CrossMab technology are widely applied to enhance secretion, enforce correct heavy- and light-chain pairing, and tailor Fc effector functions [44]. The choice of expression system directly affects product quality, cost, and scalability. Mammalian cells such as CHO and HEK293 remain the industry gold standard, producing antibodies with human-like post-translational modifications and optimal pharmacokinetic properties, though they entail high production costs and long culture times [54,55]. Yeast systems (e.g., *Pichia pastoris*) enable rapid, high-yield, and cost-efficient expression, but often generate non-human glycosylation patterns that can compromise efficacy or safety [56,57]. Bacterial systems such as *E. coli* provide fast and inexpensive expression, yet lack the ability to glycosylate proteins and frequently lead to misfolding or inclusion body formation [58,59]. Plant-based platforms offer scalability, low risk of contamination, and customizable glycoengineering, but currently face regulatory and reproducibility challenges [60,61]. Finally, cell-free expression systems facilitate rapid prototyping and are advantageous for difficult-to-express constructs, though scalability and cost remain major limitations [61,62].

While hybridoma technology offers a stable source of monoclonals, its repertoire diversity is limited. In contrast, phage and yeast display libraries enable the selection of large antibody repertoires and allow affinity maturation under controlled conditions. Ongoing clinical development efforts are primarily directed toward two key strategic approaches: (1) simultaneous blockade of oncogenic pathways (e.g., EGFR/MET) or HER2/Claudin 18.2) to overcome resistance mechanisms [7,63,64,65,66], and (2) immune cell redirection (e.g., CD3-based T-cell engagers) to enhance antitumor immunity [67,68,69,70]. Preclinical and early-phase clinical data demonstrate superior tumor selectivity, enhanced immune synapse formation, and improved penetration in the dense stroma characteristic of GI malignancies [71,72,73]. Particularly promising are BsAbs targeting HER2 and CLDN18.2 in gastric cancer, which exhibit synergistic activity while minimizing on-target/off-tumor toxicity [74]. Furthermore, the modular engineering of BsAbs allows for customized configurations (e.g., IgG-like vs. smaller scFv formats) optimized for specific clinical scenarios, the ability to flexibly design and combine these structural modules highlights the clinical versatility of bsAb platforms [75,76]. While challenges remain—particularly in managing cytokine release syndrome (CRS) and refining patient stratification strategies [77,78]—BsAbs represent a paradigm shift in the treatment of gastrointestinal cancers. Their dual specificity enables precise tumor targeting while simultaneously modulating immune activity, thus bridging the divide between traditional targeted therapies and immunotherapies. Emerging clinical and translational data support the integration of predictive algorithms for CRS risk, immune signature–based patient profiling, and cytokine pathway interference to enhance the safety and effectiveness of BsAb platforms [78,79].

**Figure 2:**
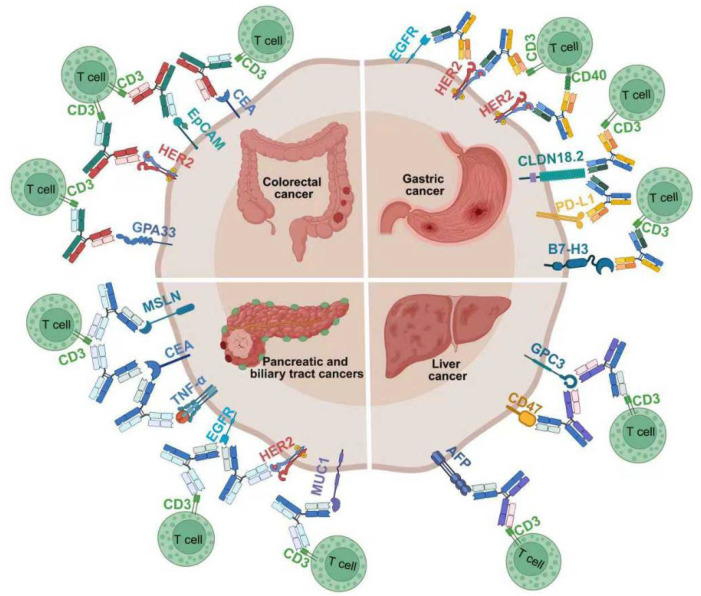
Classical molecular platforms of BsAbs in the therapy of major gastrointestinal cancer. This figure illustrates the principal mechanisms and major targets by which BsAbs exert their antitumor effects in gastrointestinal malignancies. The classical mechanisms of BsAbs action, including T-cell redirection through CD3 engagement, dual signaling blockade targeting oncogenic pathways, TME modulation. Meanwhile, it summarizes the therapeutic strategies employed across different GI cancer types, highlighting key molecular targets and representative clinical candidates. The figure provides an integrated framework for understanding the diverse mechanisms and clinical applications of BsAbs in GI oncology, facilitating the rational design of next-generation immunotherapeutic approaches. Abb: cluster of differentiation (CD), carcinoembryonic antigen (CEA), epithelial cell adhesion molecule (EpCAM), human epidermal growth factor receptor 2 (HER2), glycoprotein A33 (GPA33), mesothelin (MSLN), tumor necrosis factor-α (TNF-α), epidermal growth factor receptor (EGFR), Mucin 1 (MUC1), alpha-fetoprotein (AFP), glypican (GPC), claudin (CLDN).

This review aims to offer a comprehensive and integrative perspective on the emerging role of BsAbs in the therapeutic landscape of gastrointestinal (GI) cancers, highlighting their potential to transcend the limitations inherent to conventional mAb therapies. We begin with an overview of the epidemiology and therapeutic challenges associated with GI cancers, followed by a detailed examination of the classifications, formats, and mechanistic principles underlying BsAb design. Thereafter, we systematically summarize the main targets of BsAb application across major GI cancer subtypes, including colorectal, gastric, pancreatic, biliary, esophageal, and hepatic malignancies (Fig. 2), and focuses on translational research and clinical trials, highlighting recent advances in efficacy, safety, and therapeutic optimization. Meanwhile, we would discuss prevailing challenges—including CRS, antigenic variability, immune suppressive microenvironments. Finally, we review BsAbs combination strategies and future innovations, including combination therapy with chemotherapy and immune checkpoint inhibitors (ICIs), trispecific antibody, and AI-driven antibody engineering. Finally, the review concludes with a synthesis of key insights and outlines future directions for research, clinical application, and interdisciplinary collaboration in the field.

## 2 BsAbs in Gastrointestinal Cancer Therapy: Current Progress

### 2.1 Colorectal Cancer (CRC)

CRC ranks as the third most diagnosed malignancy and the second leading cause of cancer-related mortality globally, with over 1.9 million new cases and 935,000 deaths estimated in 2023 [80,81]. Despite advances in surgical resection and chemotherapeutic regimens, the prognosis for patients with metastatic CRC (mCRC) remains poor, largely due to treatment resistance, tumor heterogeneity, and the immunosuppressive microenvironment [82,83,84]. Durable responses are rarely achieved with standard therapies, highlighting the urgent need for novel, immune-modulating approaches such as BsAbs.

#### 2.1.1 CEA-Based Cibisatamab for CRC

**Figure 3:**
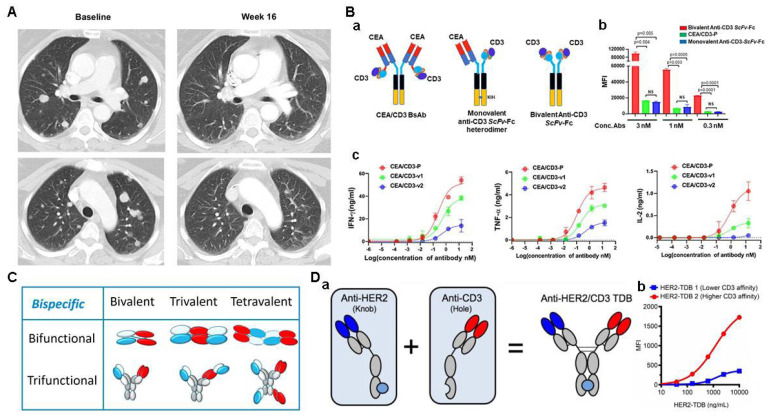
Overview of BsAbs platforms under development for CRC, illustrating diverse structural formats including bivalent, trivalent, tetravalent, bifunctional, and trifunctional configurations targeting key tumor antigens such as CEA, EpCAM, HER2, and GPA33. (**A**) Imaging assessments from a patient diagnosed with microsatellite-stable colorectal cancer (MSS-CRC) demonstrated radiological tumor reduction after administration of cibisatamab (160 mg) together with atezolizumab (1200 mg). Copyright © 2024 from Springer Nature [89]. (**B**) (**a**) depicts the t-TCE structural design of CEA × CD3 BsAbs (left), alongside a diagram of the monovalent anti-CD3 ScFv-Fc heterodimer configuration (center) and the bivalent anti-CD3 ScFv-Fc arrangement (right). (**b**) Differences in cell affinity between the monovalent and bivalent CEA × CD3 BsAbs. NS, no significance. (**c**) Quantitative analysis of IFN-γ, TNF-α, and IL-2 production by peripheral blood mononuclear cells in the presence of CEA/CD3 BsAbs. Copyright © 2021 from Oxford University Press [92]. (**C**) Bispecific antibodies can be multi-valent to increase binding strength, and those with an Fc domain recruiting macrophages are termed “trifunctional”, such as catumaxomab. Copyright © 2018 Elsevier Ltd. [102]. (**D**) (**a**) Structure of anti-HER2/CD3 TDB. (**b**) Cell affinity to human CD8^+^ cells. Copyright © 2020 from American Society for Clinical Investigation [107].

The clinical translation of BsAbs in CRC has largely focused on a defined set of tumor-associated antigens that facilitate selective targeting and immune engagement. CEA remains the most extensively validated target, particularly in microsatellite-stable (MSS)-CRC, a subtype traditionally resistant to checkpoint blockade. The most advanced BsAbs CEA × CD3 (cibisatamab), a bispecific T cell engager (TCE) that simultaneously targets CEA on tumor cells and CD3 on T lymphocytes [85], has shown potent T-cell–mediated cytotoxicity in preclinical models and has progressed through multiple phase I/II trials in MSS-CRC [86,87,88]. In comparative context, cibisatamab has demonstrated more favorable clinical outcomes in MSS-CRC, with an overall response rate (ORR) of 28% (Fig. 3A) when combined with atezolizumab [89]. Unlike MT111, which exhibited rapid clearance (t_1/2_ is approximately 4 h) and high immunogenicity (49% ADA incidence) [90], cibisatamab benefits from an optimized 2:1 CEA:CD3 valency design that enhances tumor avidity while limiting systemic T-cell activation. These findings support its potential in combinatorial immunotherapy strategies for MSS-CRC, while the overactivation of immune cells induced CRS and resistance associated with heterogeneous CEA expression remain great concerns [91,92].

#### 2.1.2 CEA-Based MT111 for CRC

Preclinical studies of MT111 (also known as AMG 211 or MEDI-565) has demonstrated potent cytotoxic activity against CEA-positive colorectal cancer (CRC) cell lines and in patient-derived xenograft (PDX) models [93]. PDX models serve as a robust preclinical platform for studying tumor progression and developing anticancer therapies [94], including those derived from chemotherapy-resistant metastatic CRC patients. Notably, MT111 exhibited significant antitumor effects at concentrations as low as 1 ng/mL [93]. *In vivo*, MT111 selectively inhibited tumor growth in CEA-positive LS174T colon cancer xenografts, but not in CEA-negative HeyA8 ovarian models. Notably, its antitumor efficacy was independent of key oncogenic mutations (KRAS, BRAF, PTEN, PIK3CA, TP53), suggesting broad applicability across molecular subtypes [90]. In a phase I trial of 39 patients with advanced gastrointestinal adenocarcinomas, MT111 demonstrated a manageable safety profile at a maximum tolerated dose of 5 mg, despite dose-limiting toxicities (DLTs) and antidrug antibody development in a subset of patients. While no objective responses were observed, stable disease was achieved in 11 cases, suggesting potential clinical benefit in selected individuals [95]. Despite initial efficacy, the development of catumaxomab and MT111 has been hindered by concerns over off-target toxicity and antigen expression in healthy tissues. Notably, catumaxomab was withdrawn from the market in 2017 due to commercial viability concerns and its unfavorable toxicity profile, representing an early cautionary tale in BsAb development [96]. Similarly, the discontinuation of MT111 development after Phase I trials highlighted the challenges of achieving meaningful clinical responses with first-generation T-cell engagers in solid tumors [97]. These failures collectively underscore the unique challenges of BsAb development in GI cancers: the immunosuppressive TME, heterogeneous antigen expression, and physical barriers to T-cell infiltration all contribute to the gap between promising preclinical activity and clinical translation. A novel trivalent TCE (t-TCE) format with monovalent, affinity-reduced CD3 binding paired with bivalent CEA targeting demonstrated the preserved cytotoxic activity against CEA-expressing tumors while substantially mitigated CRS risk (Fig. 3B) [92]. The optimization of CD3 variants (CD3-v1 or -v2) established a candidate approach to address the CRS challenge in CD3-based bispecific TCB in CRC therapy. Similarly, another comparative analysis of fragment-based and IgG-based architectures with differential valencies (anti-CEA F4/anti-CD3ε 2C11) revealed that the 2 + 1 configuration—combining a bivalent CEA-targeting diabody with a monovalent anti-CD3 scFv—demonstrated superior potency as a TCB for CRC [85]. Collectively, the findings provided evidences to modulate the therapeutic window of TCBs via module optimization.

#### 2.1.3 EpCAM-Based BsAbs for CRC

In parallel, other antigens including EpCAM have been explored in T-cell engaging BsAbs formats such as catumaxomab, MT110 and M701 et al. [98,99,100,101] Catumaxomab, the first BsAb approved for clinical use, was indicated for the treatment of malignant ascites associated with EpCAM-positive solid tumors (Fig. 3C) [102]. It consists of two Fab arms that simultaneously target the EpCAM on tumor cells and the CD3 receptor on T cells. Additionally, its functional Fc domain is capable of engaging innate immune effector cells—including macrophages, dendritic cells, and natural killer (NK) cells—via high-affinity binding to activating FcγRI and FcγRIII receptors, while sparing the inhibitory FcγRII receptor predominantly expressed on B cells [103]. Solitomab (also known as MT110 or AMG 110) is a bispecific T-cell engager (BiTE^®^) antibody construct designed for the immunotherapy of relapsed/refractory EpCAM-positive solid tumors [99]. Preclinical studies have shown solitomab’s promising anti-tumor activity across several EpCAM-positive cancer types, including ovarian, uterine, and colon cancers, by inducing robust T-cell activation, proliferation, and direct tumor cell killing. However, in a Phase 1 dose-escalation clinical trial involving patients with refractory solid tumors, solitomab faced significant challenges regarding its tolerability [99].

#### 2.1.4 HER2-Based BsAbs for CRC

Beyond CEA and EpCAM, HER2-overexpressing CRC subtypes, although rare, present actionable opportunities for HER2 × CD3 T cell engagers. Preclinical investigations have demonstrated the therapeutic potential of HER2 × CD3 BsAbs in HER2-expressing CRC, particularly in tumors resistant to conventional HER2-targeted therapies [104]. These BsAbs efficiently mediate T-cell dependent cytotoxicity both *in vitro* and in xenograft models, and their efficacy is retained even in resistant subclones [105,106]. Optimization of binding affinities and structural design has further enhanced their tumor selectivity and safety profile (Fig. 3D), underscoring their promise in overcoming immune resistance in HER2-positive CRC [107]. Novel CRC-specific antigens such as GPA33 are also under investigation; a tetravalent BsAb targeting GPA33 and CD3 has demonstrated potent T cell–mediated cytotoxicity in preclinical models [108]. Additional bispecific platforms have targeted EGFR and HER2 in tandem, aiming to inhibit compensatory signaling pathways that drive resistance to EGFR inhibition alone [109]. CrossMab and DART formats enable such dual-receptor blockade with improved pharmacokinetics and structural stability [110].

Collectively, the development of BsAbs in colorectal cancer (Table 1) has evolved from early proof-of-concept targeting well-characterized antigens to refined, structure-optimized platforms capable of modulating both efficacy and safety profiles. Advances in molecular engineering—such as affinity-tuned CD3 binding, 2 + 1 valency formats, and fragment-based constructs—have expanded the therapeutic window of T cell engagers, particularly in immunologically “cold” tumors like MSS-CRC. While clinical translation of early agents such as catumaxomab and MT111 was limited by off-tumor toxicity and immunogenicity, newer constructs like cibisatamab and HER2 × CD3 BsAbs show improved selectivity and potential synergy with immune checkpoint blockade. Furthermore, the exploration of alternative targets (e.g., GPA33, EGFR-HER2 pairs) and the integration of bispecifics into rational combination regimens mark a promising direction toward overcoming resistance mechanisms and enhancing durable responses. As ongoing clinical trials and translational studies continue to refine antigen selection, construct design, and patient stratification strategies, BsAbs are poised to become an integral component of precision immunotherapy in colorectal cancer.

**Table 1:** Summary of advanced BsAbs in colorectal cancer therapy.

BsAbs	Preclinical Platform	Effector Cell	Clinical Trials	Reference
Anti-CEA × CD3	RG7802 RO6958688	T cell	NCT02324257	[88]
	Cibisatamab	T cells	NCT02324257 NCT02650713	[89]
	MT111 (AMG 211 or MEDI-565)	T cells	NCT01284231	[95]
	Other variants	T cells	—	[85,92]
Anti-EpCAM × CD3	Catumaxomab	T cells, macrophages, dendritic cells, and NK cells	—	[103]
	Solitomab (MT110 or AMG 110)	T cell	—	[99]
Anti-HER2 × CD3	—	T cell	—	[104,105,106]
Anti-GPA33 × CD3	—	T cell	—	[108]
Anti-EGFR × HER2	—	T cell	—	[109]

### 2.2 Gastric Cancer (GC)

GC remains a major contributor to global cancer mortality, ranking as the fifth most common malignancy and the third leading cause of cancer-related deaths worldwide, with over one million new cases annually [111,112]. The burden is disproportionately high in East Asia—particularly in China, Japan, and Korea—where dietary risk factors, *Helicobacter pylori* infection, and limited screening contribute to elevated incidence and late-stage diagnosis [113,114]. Despite advances in systemic chemotherapy, HER2-targeted agents such as trastuzumab, and ICIs like nivolumab, the prognosis for advanced GC remains dismal, with median overall survival rarely exceeding one year [115,116]. Major obstacles include the extensive molecular heterogeneity of GC—reflected in variable HER2 amplification, microsatellite instability (MSI), and Epstein–Barr virus (EBV) subtypes—and the immune-evasive TME [116,117]. These factors collectively undermine durable responses to current therapeutic modalities and highlight an urgent need for more precise, immune-activating strategies [111,115,117].

#### 2.2.1 HER2-Based BsAbs for GC

**Figure 4:**
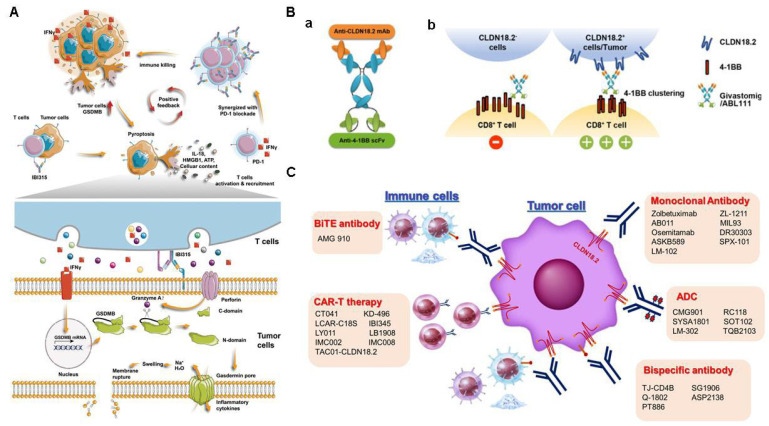
Schematic overview of BsAbs platforms under development for GC, illustrating diverse therapeutic strategies targeting key molecular antigens including HER2, CLDN18.2, and PD-L1 through various mechanisms such as T-cell redirection, co-stimulatory receptor engagement (4-1BB), and immune checkpoint modulation. (**A**) Diagram illustrating the immune checkpoint modulation pathway through which IBI315 induces pyroptotic death in HER2-expressing tumor cells. Copyright © 2023 from Wiley-VCH GmbH [124]. (**B**) (**a**) Structure and (**b**) action mechanism of Givastomig/ABL111 BsAb. Copyright © 023 from BMJ Publishing Group Ltd. [128]. (**C**) BsAbs and other therapeutic platforms rationally designed based on Claudin18.2 overexpression. Copyright © 2023 from MDPI [132].

The development of BsAbs in GC has initially centered around HER2, a well-characterized oncogenic driver in a subset of GC [117,118]. Among the most notable HER2-directed BsAbs is ABP-102 (CT-P72), a CD3 × HER2 T-cell engager engineered with Fc silencing and affinity-optimized CD3 binding to reduce CRS. In preclinical evaluations, ABP-102 demonstrated selective cytotoxicity toward HER2-high GC cells, while sparing HER2-low normal tissues, reflecting an improved therapeutic window. Meanwhile, ABP-102/CT-P72 achieved approximately twice the antitumor efficacy relative to a runimotamab analogs. Furthermore, dose-escalation schedules in preclinical models have shown manageable safety profiles, laying the groundwork for upcoming first-in-human trials [119]. In addition, GenSci139, a BsAbs-drug conjugate (BsADC) targeting EGFR and HER2, is under investigation for HER2-amplified GC. Unlike traditional BsAbs, GenSci139 delivers a cytotoxic payload via internalization after dual-antigen engagement, enhancing tumor cell specificity. In GC PDX, GenSci139 significantly outperformed its parental HER2 monoclonal antibody, particularly in tumors with moderate HER2 expression, a population that typically shows limited response to trastuzumab [120]. In addition to HER2 × CD3 constructs, several dual-targeting BsAbs—such as KN026 (HER2 × HER2), CD40 × HER2, and IBI315 (PD-1 × HER2)—have demonstrated encouraging activity in clinical studies. These agents operate through distinct yet complementary mechanisms, including blockade of compensatory receptor signaling (HER2 inhibition) [121,122], enhancement of antigen presentation and T cell priming (via CD40 engagement) [123], and concurrent immune checkpoint modulation (Fig. 4A) [124]. Notably, such dual-specific constructs have shown potential to overcome resistance to conventional HER2-targeted therapies and broaden therapeutic applicability across heterogeneous HER2-amplified GC populations.

#### 2.2.2 CLDN18.2 × 4-1BB BsAbs for GC

Another major focus has been CLDN18.2, a tight-junction protein aberrantly expressed on malignant gastric epithelium but minimally on normal tissues. CLDN18.2 × CD3 BsAbs such as AMG910, QLS31905, ZWB67 have demonstrated highly selective T-cell engagement and potent cytotoxicity against CLDN18.2-positive tumor cells in preclinical models [125,126,127]. ZWB67 induced robust T cell activation and tumor cell lysis in a CLDN18.2 expression–dependent manner. Co-culture assays confirmed elevated interferon-γ (IFN-γ) and TNF-α production, along with effective killing of CLDN18.2-positive cells, while sparing CLDN18.2-negative controls. In humanized xenograft models, ZWB67 significantly suppressed tumor growth and enhanced CD8^+^ T cell infiltration without off-tumor toxicity [127]. Givastomig (TJ-CD4B/ABL111) is a first-in-class BsAbs targeting CLDN18.2 and 4–1BB, designed to activate T cell through antigen-dependent co-stimulation within the TME (Fig. 4B) [128,129]. Unlike CD3-based T cell engagers, it offers enhanced immune specificity with reduced systemic toxicity. *In vitro*, Givastomig induced CLDN18.2-dependent T cell activation and effective tumor lysis, including bystander killing of adjacent CLDN18.2-negative cells. Combined with standard chemotherapies (e.g., FOLFOX, paclitaxel), it further enhanced antitumor responses. In a GC PDX model, triple therapy with Givastomig, FOLFOX, and nivolumab yielded superior tumor growth inhibition (40% vs. 8%) and increased T cell infiltration [130]. In an early-phase clinical trial of Givastomig, the agent demonstrated a favorable safety profile with no DLTs observed up to 15 mg/kg and no grade ≥ 4 treatment-related adverse events. Preliminary antitumor activity was observed in patients with CLDN18.2-positive GC, including confirmed partial responses at multiple dose levels, with clinical benefit seen across a broad range of CLDN18.2 expression (11–100%) [129]. Building on this strategy, OriA362, a CLDN18.2 × 4-1BB BsAbs with optimized antigen and co-stimulatory binding affinities, demonstrated superior preclinical efficacy in both low- and high-CLDN18.2-expressing tumor models. In human 4-1BB transgenic mice, OriA362 outperformed both Givastomig and the CLDN18.2 monoclonal antibody IMAB362, achieving complete tumor regression at low doses and inducing durable immunologic memory evidenced by tumor rejection upon rechallenge [131]. These findings highlight OriA362’s potential to overcome antigen heterogeneity while sustaining long-term antitumor immunity [131].

#### 2.2.3 CLDN18.2 × PD-L1 BsAbs for GC

The combination of PD-L1 ICIs with CLDN18.2-targeted therapies also holds promising therapeutic potential. Q-1802 is a novel humanized BsAb designed to simultaneously target CLDN18.2 and PD-L1, integrating tumor-specific antigen recognition with immune checkpoint blockade. This dual-targeting strategy is particularly suited to the immunosuppressive microenvironment of GC, where CLDN18.2 is selectively overexpressed in malignant cells (Fig. 4C) and PD-L1 contributes to T cell exhaustion [132,133]. Compared to PD-L1 inhibitor monotherapy, the CLDN18.2 × PD-L1 bispecific approach offers several distinct advantages: (1) localized immune activation at the tumor site through CLDN18.2-mediated targeting, reducing systemic immune-related adverse events; (2) enhanced tumor specificity through dual-antigen recognition; and (3) potential activity in tumors with heterogeneous PD-L1 expression, as CLDN18.2 binding provides an anchor for immune engagement. Preclinical studies have shown that Q-1802 exerts potent antitumor effects through T-cell reactivation and direct tumor targeting, even in models with heterogeneous CLDN18.2 expression. The bispecific format allows for localized PD-L1 inhibition at the tumor site, potentially reducing systemic toxicity often observed with conventional anti PD-L1 therapies [132,134]. In an ongoing Phase 1a/1b clinical trial (NCT04856150), Q-1802 is being evaluated in patients with advanced GI cancers, including gastric and gastroesophageal junction adenocarcinoma. Interim data indicate that Q-1802 demonstrates a favorable safety profile, with manageable adverse events and preliminary signals of clinical activity. Another Phase Ib/II trial (NCT05964543) of Q-1802 in combination with chemotherapy (XELOX) in patients with treatment-naive advanced gastric/gastroesophageal junction cancer revealed a significant trend: among patients with high CLDN18.2 expression (moderate-to-strong membranous staining in ≥70% of tumor cells), the ORR was 70.3% (26/37). Notably, in patients with concurrent PD-L1 expression (CPS ≥ 5) and high CLDN18.2 expression, the ORR reached 81.8% (9/11) [135]. This analysis indicates that even in the presence of PD-L1 expression heterogeneity (with CPS ≥ 5 as the enrollment threshold), Q-1802 maintains potent antitumor activity through CLDN18.2-mediated anchoring, provided that the tumor exhibits high CLDN18.2 expression. Partial responses have been observed in several CLDN18.2-positive GC patients, including those previously resistant to checkpoint inhibitors [133,136,137]. Notably, Q-1802 represents a first-in-class BsAb combining tumor specificity and immune modulation in a single molecule. This makes it particularly promising in checkpoint-refractory or low-immunogenic tumors, and it may offer a more focused immunotherapy approach than PD-1/PD-L1 inhibitors alone, especially in Asian populations with a higher prevalence of CLDN18.2-positive gastric tumors [138,139,140].

#### 2.2.4 CLDN18.2 × HER2 BsAbs for GC

To address the molecular complexity of gastric tumors, combinatorial designs CLDN18.2 × HER2 BsAbs have been engineered to simultaneously target co-expressing antigens. Preclinical construct employing this CLDN18.2 × HER2 BsAbs (HC-2G4S) exhibited enhanced immune effector recruitment and tumor cell lysis, particularly in heterogenous tumors expressing both markers [74].

#### 2.2.5 Other BsAbs for GC

Beyond established targets above, several alternative tumor-associated antigens are under investigation for BsAb–based immunotherapy in GC (Table 2). A CD3-engaging BsAb targeting Lewis Y (m3s193) demonstrated potent T cell activation and tumor clearance in preclinical GC models, highlighting the potential of glycan-targeted immunotherapy [141]. Similarly, the B7-H3 × CD3 BsAb CC-3 induced robust T cell activation, cytokine release, and memory formation, showing strong antitumor activity in gastric, hepatic, and pancreatic cancer models [142]. In addition, an EGFR × CD3 BsAb enhanced the cytotoxicity of cytokine-induced killer (CIK) cells against EGFR-positive GC both *in vitro* and *in vivo* [143]. These findings support Lewis Y, B7-H3, and EGFR as emerging BsAb targets in GC, especially for tumors lacking conventional biomarkers or resistant to checkpoint inhibitors.

**Table 2:** Summary of advanced BsAbs in gastric cancer therapy.

BsAbs	Preclinical Platform	Effector Cell/Functions	Clinical Trials	Reference
Anti-HER2 × CD3	ABP-102 (CT-P72)	T cell	—	[119]
Anti-EGFR × HER2	GenSci139	Inhibition of EGF-induced signaling and cell proliferation	—	[120]
Anti-HER2 × HER2	KN026	HER2 inhibition	NCT03925974	[121,122]
Anti-CD40 × HER2	—	Targeting CD40 to restore the ubiquitination level of CCL2-ZC3H12A-TRAF6/3 signaling axis	—	[123]
Anti-PD-1 × HER2	IBI315	Triggering gasdermin B (GSDMB)-mediated pyroptosis	—	[124]
Anti-CLDN18.2 × CD3	AMG910	T cell	NCT04260191	[125]
	QLS31905	T cell	NCT05278832	[126]
	ZWB67	T cell	—	[127]
Anti-CLDN18.2 × 4–1BB	Givastomig (TJCD4B/ABL111)	T cell	NCT04900818	[128,129,130]
	OriA362	Eliciting TAA-dependent 4-1BB activation	—	[131]
Anti-CLDN18.2 × PD-L1	Q-1802	Blocking PD-1 signaling and activating innate immunity	NCT04856150	[136,144,145]
Anti-CLDN18.2 × HER2	HC-2G4S	Antibody-dependent cell-mediated cytotoxicity	—	[74]
Anti-Lewis Y × CD3	m3s193	T cell	—	[141]
Anti-B7-H3 × CD3	CC-3	T cell	—	[142]
Anti-EGFR × CD3	—	Cytokine-induced killer (CIK) cells	—	[143]

### 2.3 Pancreatic and Biliary Tract Cancer (PC and BTC)

PC and BTC represent a group of highly aggressive malignancies with poor prognosis. PC is the 12th most common cancer globally, but ranks as the 7th leading cause of cancer-related mortality, accounting for approximately 466,000 deaths annually [146]. The incidence varies geographically, with higher rates observed in developed countries, particularly in North America and Europe. Risk factors include smoking, obesity, chronic pancreatitis, diabetes mellitus, and hereditary syndromes (e.g., BRCA mutations, Lynch syndrome) [147]. BTCs, comprising intrahepatic cholangiocarcinoma (iCCA), extrahepatic cholangiocarcinoma (eCCA), and gallbladder cancer (GBC), exhibit distinct epidemiological patterns. Globally, BTCs account for ~3% of gastrointestinal malignancies, with significant regional variations [148]. Gallbladder cancer is more prevalent in South America and Asia (particularly India and Chile), linked to gallstone disease and chronic infections (e.g., *Salmonella typhi*). Cholangiocarcinoma incidence has risen in Western countries, possibly due to improved diagnostics and increasing metabolic syndrome-related liver diseases (e.g., non-alcoholic fatty liver disease) [149]. Both pancreatic and biliary cancers are typically diagnosed at advanced stages, contributing to their 5-year survival rates of <10% [150]. At the molecular level, BsAbs overcome immune exclusion in pancreatic and biliary cancers through multiple mechanisms: bypassing MHC restriction despite downregulated MHC class I expression [151,152]; facilitating immune synapse formation despite dense desmoplastic stroma [153]; inducing local pro-inflammatory cytokine secretion (IFN-γ, TNF-α) that reshapes the immunosuppressive TME [151,154]; and simultaneously targeting stromal components or immunosuppressive ligands that limit T-cell infiltration [155,156]. The development of BsAbs in BTCs has primarily focused on targeting tumor-associated antigens (TAAs) that are overexpressed in these malignancies, including MSLN, CEA, GPC1, and CLDN18.2.

#### 2.3.1 MSLN-Based BsAbs for PC and BTC

MSLN, a glycosylphosphatidylinositol (GPI)-anchored surface protein, is overexpressed in approximately 80% of pancreatic ductal adenocarcinomas (PDAC) and 30–50% of cholangiocarcinomas (CCA), while exhibiting minimal expression in normal tissues—rendering it an attractive and selective target for BsAb-based immunotherapy [157,158,159]. The IgG-like BsAbs MG1122 has demonstrated robust antitumor activity against MSLN-positive PDAC through effective redirection of CD3^+^ T cells, inducing tumor-specific cytotoxicity as evidenced by caspase-3/7 activation and upregulation of canonical T cell activation markers (e.g., NFAT signaling, CD25, CD69) [160,161,162]. Notably, MG1122 retains potent cytotoxic function in the presence of soluble MSLN, overcoming a key mechanism of immune evasion that hampers many MSLN-targeted therapies [160]. These findings support MG1122 as a promising candidate for clinical translation, with its full-length IgG format offering favorable pharmacokinetic properties. Recent molecular engineering advances have yielded optimized BsAb constructs such as the MSLN HLE BiTE^®^, which achieves picomolar-level cytotoxicity against chemo-resistant PDAC via high-affinity dual engagement of CD3 and MSLN [163]. It maintains target specificity, confirmed in MSLN-knockout models, and benefits from half-life extension technologies to improve *in vivo* stability [163]. In comparative xenograft studies, HPN536, an MSLN × CD3 BsAb, exhibited superior efficacy in the HPAFII pancreatic tumor model—achieving complete responses at a dose of 20 μg/kg, five times lower than that required in ovarian (TOV21G) and NSCLC (NCI-H292) models—highlighting its tumor-type–specific potency [164]. Further innovations include NM28-2746, a bivalent T cell engager that discriminates tumor cells based on antigen density, thereby enhancing tumor selectivity while mitigating risks associated with soluble antigen interference and on-target/off-tumor toxicity. Further combination strategies have also shown promise: co-administration of NM28-2746 with NM21-1480, a bifunctional PD-L1/4-1BB modulator, produced synergistic antitumor effects [165]. Additionally, combinatorial activation of innate-like T cell subsets—specifically NKT and γδ T cells—via α-GalCer and zoledronate, further enhanced the efficacy of MSLN × CD3 BsAbs without increasing toxicity [166]. These results underscore the potential of expanding beyond conventional αβ T cell–based approaches to improve the therapeutic index in PDAC immunotherapy.

#### 2.3.2 CEA-Based BsAbs for PC and BTC

CEA is expressed in >90% of PCs and approximately 50% of iCCA [167,168,169]. MT111 have demonstrated dose-dependent pharmacokinetics with rapid clearance (t_1/2_ is approximately 4 h) in patients with advanced GI cancers, though clinical efficacy was limited to disease stabilization at tolerable doses (MTD 3 mg). While manageable with dexamethasone premedication, DLTs (hypoxia, CRS) and immunogenicity highlight the challenges of first-generation T-cell engagers in solid tumors [95]. Another CEA TCB (RO6958688/RG7802) adopted a novel 2:1 asymmetric IgG-based configuration, featuring bivalent binding to CEA and monovalent binding to CD3. This stoichiometric design allows for enhanced tumor cell avidity while limiting excessive CD3 engagement, thereby reducing the risk of systemic T cell overactivation and CRS. In preclinical models of CEA-positive pancreatic adenocarcinoma, notably the BxPC-3 cell line, CEA TCB induced robust and target-specific cytotoxicity mediated by peripheral blood mononuclear cells (PBMCs) [170]. A BsAbs targeting CEA and human TNF-α was engineered to localized immunomodulation by directing TNF-α to CEA^+^ pancreatic tumor sites [171]. CEA × TNF-α BsAbs amplifies the radiosensitizing effects of TNF-α and prolongs tumor control duration *in vivo* and further circumvents the dose-limiting systemic toxicities traditionally associated with TNF-α therapy.

#### 2.3.3 EGFR-Based BsAbs for PC and BTC

EGFR is overexpressed or dysregulated in a significant proportion of PDAC cases and 67–100% of biliary cancers, making it an attractive therapeutic target [172,173]. Despite the modest clinical benefit of EGFR-targeted monoclonal antibodies (e.g., cetuximab) and tyrosine kinase inhibitors in PDAC [174,175,176], recent efforts have focused on EGFR × CD3 BsAbs, which aim to redirect cytotoxic T cells toward EGFR^+^ tumor cells, bypassing the limitations of EGFR pathway inhibition alone [152]. In a phase I/II trial, infusions of activated T cells armed with an EGFR × CD3 BsAbs were well tolerated and induced robust immune responses in patients with advanced PC [177]. The therapy resulted in a median overall survival of 31 months and durable disease control in several patients, including two complete responses following chemotherapy. These findings support EGFR-targeted CD3 BsAb-armed T cell therapy is safe, immune-stimulatory, and may improve survival in advanced PC, especially when used as part of a multimodal regimen. Another representative platform, T-MATE (TME activated therapeutics), has engineered pH-sensitive CD3-engaging BsAbs that become functionally active specifically within the acidic TME. Among the targets investigated in this format are EGFR, TROP2, and FOLR1, all of which are frequently expressed in solid tumors including PDAC. Preclinical data showed that EGFR × CD3 T-MATE BsAbs induced potent T cell-mediated cytotoxicity in PC cell lines and were well tolerated *in vivo* due to their tumor-selective activation mechanism [178]. Using a 2 + 2 IgG(L)-scFv tetravalent format, researchers designed BsAbs that simultaneously engage CD3^+^ T cells and tumor cells via dual Fab arms derived from cetuximab (anti-EGFR) and trastuzumab (anti-HER2), integrated using a knob-into-hole heterodimerization strategy [109]. Preclinical evaluations showed that EGFR × HER2 BsAbs, alongside their homodimeric counterparts (EGFR × EGFR, HER2 × HER2), exhibited picomolar-level avidity and cytotoxicity against PDAC cell lines. Functionally, the EGFR × HER2 BsAb demonstrated selective and potent tumor ablation only in EGFR^+^HER2^+^ double-positive PDAC xenografts, but not in single-antigen knockout models, suggesting a tumor-specific dual-targeting advantage. Building upon the strategy of dual ErbB receptor targeting in PC, the BiXAb^TM^ tetravalent antibody platform offers an alternative and innovative design that further enhances the therapeutic potential of BsAbs against EGFR, HER2, and HER3 [32]. In this approach, BsAbs were constructed using rational epitope pairing and screened against a variety of functional benchmarks, including receptor binding, intracellular signaling suppression, tumor cell proliferation, apoptosis induction, and immune effector engagement (e.g., ADCC and CDC). Out of 30 candidates, 3Patri-1Cetu-Fc, 3Patri-1Matu-Fc, and 3Patri-2Trastu-Fc emerged as lead BsAbs with superior performance both *in vitro* and *vivo* assays. These antibodies exhibited robust inhibition of phosphorylation-driven signaling, effective induction of tumor cell apoptosis, and enhanced immune effector recruitment. Most notably, in preclinical xenograft models of PC, these BsAbs achieved deep tumor penetration-overcoming the dense desmoplastic stroma characteristic of PDAC and produced significant tumor growth suppression compared to parental antibodies and their combinations.

#### 2.3.4 CLDN18.2-Based BsAbs for PC and BTC

CLDN18.2-targeting BsAbs also represent a promising therapeutic strategy for PC, given the frequent overexpression of this tight junction protein in malignant pancreatic epithelium. An anti-CLDN18.2 antibody drug conjugates (ADCs) and CD3-bispecific constructs demonstrated potent *in vitro* cytotoxicity (IC50 range: 0.07–2.03 nM) against CLDN18.2+ pancreatic (BxPC3) and gastric (KATO-III) cancer lines, and significant antitumor activity was observed in PDX models of both cancer types [179].

#### 2.3.5 B7-H3-Based BsAbs for PC and BTC

Emerging targets such as B7-H3 (CD276), MUC1 and Siglec-15 (S15) have shown promise in preclinical models. B7-H3, a member of the B7-superfamily of immunoregulatory ligands, exhibits minimal expression in normal tissues but is frequently overexpressed in a variety of malignancies [180,181], especially in the pancreato-biliary subtype of ampullary cancer [182], making it attractive for BsAb design aimed at enhancing T cell infiltration. CC-3 is an IgG-based BsAbs targeting B7-H3 and CD3, designed to redirect T cells toward B7-H3–expressing tumors such as pancreatic, hepatic, and GCs. It induces robust T cell activation, cytokine secretion (IL-2, IFN-γ, perforin), and memory T cell formation, resulting in potent tumor cell lysis in preclinical models [142]. The combination therapy of B7-H3 × CD3 BsAbs with MEK inhibitor has demonstrated with amplified tumor suppression and immune infiltration [183].

#### 2.3.6 MUC1-Based BsAbs for PC and BTC

MUC1 is a transmembrane glycoprotein highly expressed and aberrantly glycosylated in PDAC and CCA [184]. SEA D227A-M × 3 diabody represents a novel recombinant MUC1 × CD3 BsAbs engineered to enhance T cell-mediated cytotoxicity against MUC1-positive malignancies such as bile duct carcinoma (BDC). Structurally, SEA D227A-M × 3 is based on a diabody format, consisting of the variable regions of anti-MUC1 and anti-CD3 antibodies, genetically fused with a mutated superantigen (staphylococcal enterotoxin A, SEA D227A) to amplify T cell activation [185]. While MUC1 remains a highly appealing tumor-associated antigen for BsAbs development, therapeutic application is hindered by its partial expression in normal tissues, soluble antigen interference, intratumoral heterogeneity, and relatively limited T cell activation. These factors underscore the need for precise epitope targeting, structural refinement, and combinatorial strategies to enhance clinical efficacy. Other Emerging targets have also advanced for BsAbs development against PC.

#### 2.3.7 S15-Based BsAbs for PC and BTC

S15, a membrane-associated immunosuppressive molecule, is consistently upregulated in PDAC and iCCA, predominantly within tumor-associated immune and stromal cells [186,187]. Its expression is mutually exclusive with PD-L1 in many contexts and is associated with T cell exclusion and immunosuppressive microenvironments, supporting its development as a novel checkpoint blockade target in gastrointestinal cancers [188,189,190]. STAB, a BsAbs featured an IgG-scFv format capable of simultaneously binding S15 on tumor cells and CD3 [191]. Preclinical investigations demonstrated that STAB induced robust T cell proliferation and activation in co-culture assays with human PBMCs and effectively mediated cytotoxic lysis of S15-positive PDAC cells (Panc-1) and NSCLC models [191]. Notably, STAB outperformed monotherapies targeting S15 or CD3 alone, and enhanced T cell infiltration while reducing stromal density, a major barrier in PDAC immunotherapy.

#### 2.3.8 GPC-1-Based BsAbs for PC and BTC

MIL-38-CD3, a novel BiTE, represents a promising immunotherapeutic approach targeting GPC-1—a heparan sulfate proteoglycan highly overexpressed in various solid tumors, including PC and prostate cancer [192,193]. This bispecific construct integrates the anti-GPC-1 scFv sequence from the clinical-stage antibody Miltuximab^®^ with the anti-CD3 scFv derived from Blinatumomab, designed to redirect T cells selectively toward GPC-1^+^ tumor cells [192]. The BiTE construct effectively induced T cell activation (CD25, CD69 upregulation) and proinflammatory cytokine secretion (TNF-α, IFN-γ) in co-culture with GPC-1–positive prostate cancer cells (PC3 and DU-145), while sparing GPC-1-low or -negative cells. Importantly, the upregulation of PD-1 on activated T cells suggests that this BiTE may benefit from combination with ICIs to counteract T cell exhaustion.

Collectively, these findings highlight a growing arsenal of BsAb targets in BTCs, including MSLN, CEA, EGFR, B7-H3, MUC1and potentially GPC-1—with therapeutic promise rooted in their ability to engage effector T cells and reshape the immune-suppressive TME (Table 3). Rational combination strategies with checkpoint blockades, innate immune modulators, or stromal-depleting agents may further enhance the clinical impact of BsAbs in these otherwise poorly immunogenic malignancie.

**Table 3:** Summary of advanced BsAbs in therapy of pancreatic and biliary tract cancers.

BsAbs	Preclinical Platform	Effector Cell/Functions	Clinical Trials	Reference
Anti-MSLN × CD3	MG1122	T cell	—	[160,161,162]
	MSLN HLE BiTE^®^	T cell	—	[163]
	HPN536	T cell	NCT03872206	[164]
	NM28-2746	T cell	—	[165]
Anti-CEA × CD3	RO6958688/RG7802	T cell	—	[170]
Anti-CEA × TNF-α	—	Antibody-dependent cell-mediated cytotoxicity	—	[171]
Anti-EGFR × CD3	—	T cell	NCT01420874 NCT02620865	[177]
Anti-EGFR × HER2/HER3	3Patri-1Cetu-Fc, 3Patri-1Matu-Fc 3Patri-2Trastu-Fc	Antibody-dependent cell-mediated cytotoxicity	—	[32]
Anti-B7-H3 × CD3	CC-3	T cell	—	[183]
Anti-MUC1 × CD3	—	T cell	—	[185]
Anti-S15 × CD3	STAB	T cell	—	[191]
Anti-GPC-1 × CD3	MIL-38-CD3	T cell	—	[192,193]

### 2.4 Liver Cancers

According to recent GLOBOCAN data, liver cancer ranks as the third leading cause of cancer-related mortality globally, while esophageal cancer ranks sixth in mortality and seventh in incidence, with notable geographic disparities in prevalence and etiology [194]. Hepatocellular carcinoma (HCC), the predominant subtype of liver cancer, is frequently associated with chronic hepatitis B or C infections and cirrhosis, and disproportionately affects populations in East and Southeast Asia and sub-Saharan Africa [195]. Meanwhile, esophageal cancer displays two major histological subtypes—squamous cell carcinoma (ESCC) and adenocarcinoma (EAC)—with ESCC more common in Asia and Africa, and EAC prevalent in Western countries [196]. Despite advances in surveillance and treatment, both cancers are often diagnosed at advanced stages and are associated with poor prognosis, underscoring the urgent need for innovative therapeutic modalities, including BsAbs-based immunotherapies [197]. Recent advancements in BsAbs engineering have enabled the targeting of novel tumor-associated antigens in esophageal and liver cancers, particularly HCC, with promising preclinical and early clinical outcomes. Several key tumor-specific antigens have emerged as attractive BsAbs targets, including GPC3, AFP, CD3, and CD47 (Table 4), offering new mechanisms for immune redirection and tumor eradication.

**Table 4:** Summary of advanced BsAbs in liver cancer therapy.

BsAbs	Preclinical Platform	Effector Cell/Functions	Clinical Trials	Reference
Anti-GPC3 × CD3	ERY974	T cell	—	[198,199,200,201,202]
Anti-GPC3 × CD47	—	Restoring phagocytosis and enhance antitumor immunity via Fc effector functions	—	[203,204,205,206]
Anti-AFP × CD3	—	—	—	[207,208]

#### 2.4.1 GPC3 Based-BsAbs for HCC

GPC3 is a highly promising target in HCC due to its selective overexpression in tumors and negligible expression in normal adult tissues [209,210,211]. BsAbs targeting GPC3 often pair it with immune cell-engaging epitopes such as CD3 or CD47, thereby maximizing both specificity and cytotoxic potential. One of the most prominent agents is ERY974, a GPC3 × CD3 bispecific T-cell engager [212,213,214]. Its structure incorporates a common light chain architecture, which enhances manufacturability and stability while maintaining precise bispecific binding. ERY974 demonstrates potent cytotoxicity in preclinical models and early-phase clinical trials, even in poorly immunogenic tumors [213]. This effect is achieved by converting immune-cold tumors into inflamed microenvironments via IFN-γ and TNF-α upregulation [215]. IgG-based heterodimeric BsAbs and BiTE (bispecific T-cell engager) constructs have demonstrated encouraging preclinical efficacy, with distinct engineering strategies employed to improve stability, specificity, and immune activation. The IgG-based GPC3 × CD3 BsAbs utilizes Knob-into-Hole mutations for heavy chain heterodimerization and Fc domain substitutions (P329G/L234A/L235A) to reduce Fcγ receptor and complement activation, while retaining favorable pharmacokinetics [198]. Functionally, it induces potent, GPC3-dependent cytotoxicity by bridging CD3^+^ T cells with GPC3^+^ tumor cells, leading to significant tumor suppression in xenograft models, but only in the presence of human effector cells—confirming tumor-specific immune redirection. An advanced XmAb^®^ heterodimeric Fc platform has enabled the development of a structurally optimized GPC3 × CD3 BsAbs in a 2 + 1 Fab_2_-scFv-Fc format, characterized by bivalent binding to GPC3 and monovalent engagement of CD3 [199]. This design aims to balance potent T cell-mediated cytotoxicity with tumor selectivity, thereby minimizing potential off-tumor toxicity. Apart from binding the heavily glycosylated C-terminal region, where epitope masking often compromises therapeutic efficacy, researchers developed Pro-12, a high-affinity humanized IgG1 antibody directed against the N-terminal 25–45 peptide of GPC3, which is more accessible and functionally relevant [202]. The engineered GPC3 × CD3 BsAbs, based on Pro-12 in CrossMab and Knob-into-Hole technologies, exhibited dual mechanisms of action: robust T-cell activation and direct modulation of oncogenic signaling pathways, specifically Wnt/β-catenin and PI3K/AKT, which are critical to HCC progression. Notably, this bispecific construct achieved functional outcomes that typically require tri-specific antibodies, highlighting its structural and therapeutic innovation.

**Figure 5:**
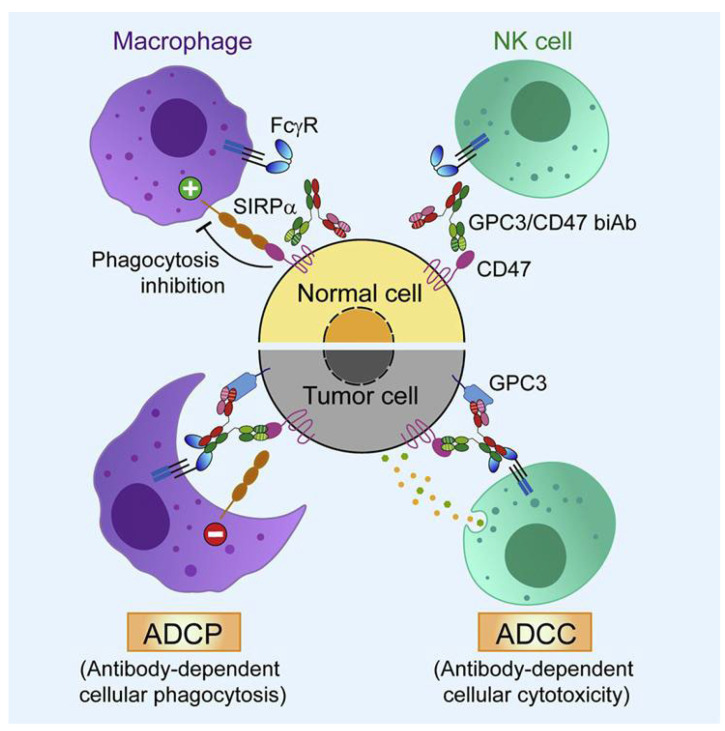
Schematic illustration of the mechanism of action of GPC3 × CD47 BsAb in HCC, demonstrating how simultaneous targeting of GPC3 on tumor cells and CD47 on macrophages enhances ADCP and ADCC while overcoming the “don’t eat me” signal-mediated immune evasion. Copyright © 2021 from The American Society of Gene and Cell Therapy [206].

In parallel, a BiTE construct was generated by fusing the scFv of humanized anti-GPC3 (clone 9F2) with that of anti-CD3 (OKT3), forming a minimal yet effective engager [216]. This format exhibits strong *in vitro* and *in vivo* T cell cytotoxicity against GPC3-positive HCC lines, with no detectable off-tumor effects due to the cancer-restricted expression of GPC3. Furthermore, the BiTE format benefits from its compact size, enhancing tumor penetration and rapid immune synapse formation [216]. An assay on six GPC3 × CD3 BsAbs for HCC, using three structural formats: knob-into-hole (KH), scFv-scFv-hFc, and scFv-hFc-scFv [200], confirmed strong GPC3-dependent killing across all BsAbs, with hYP7-KH, hYP7-OKT3-hFc, and HN3-KH being the most potent. Notably, combining hYP7-OKT3-hFc with the chemotherapeutic agent irinotecan significantly enhanced tumor suppression in multiple HCC models, suggesting that this BsAbs format, particularly when combined with chemotherapy, offers a promising strategy for controlling advanced GPC3-positive HCC. Furthermore, the integration of GPC3 × CD3 TRAB (T cell-redirecting antibody) BiTE with GPC3-specific optical imaging probes offers a novel theragnostic strategy for HCC [201]. The peptide-derived BiTE effectively activated T cells and induced cytotoxicity against GPC3^+^ HCC cells *in vitro* and in PDX models. Concurrently, the imaging probe enabled real-time, noninvasive tumor detection and monitoring of therapeutic response. This dual-modality approach highlights the potential of GPC3-targeted BiTEs for both treatment and precision imaging in HCC. Additionally, GPC3 × CD47 BsAbs have emerged to counteract innate immune suppression. CD47, a “don’t eat me” signal overexpressed in HCC, impairs macrophage- and neutrophil-mediated clearance [203,204,205]. GPC3 × CD47 BsAbs restore phagocytosis and enhance antitumor immunity via Fc effector functions (Fig. 5) [206]. This bispecific approach differs fundamentally from simple CD47 blockade: the GPC3-binding arm provides tumor-specific localization, sparing normal tissues that express CD47 but not GPC3, thereby mitigating anemia and thrombocytopenia associated with systemic CD47 inhibition. Additionally, preserved Fc effector functions engage activating Fcγ receptors on macrophages, providing synergistic pro-phagocytic signaling beyond CD47-SIRPα blockade. In murine xenograft models with hSIRPα/hCD47 humanization, these bispecifics exhibited superior tumor growth suppression compared to monotherapies and combination mAb approaches, while avoiding systemic toxicity.

#### 2.4.2 AFP Based-BsAbs for HCC

AFP, a well-established serum biomarker for HCC, has recently gained attention as a tumor-specific antigen for BsAbs-based immunotherapy [217,218,219]. Due to its re-expression in malignant hepatocytes and absence in normal adult tissues, AFP offers a favorable therapeutic window for targeted intervention. Strong preclinical evidence has illustrated that AFP as a viable target for T-cell redirection, which is a core principle behind many BsAbs designs [220]. Emerging bispecific platforms, particularly T cell engagers that target AFP and CD3, have demonstrated promising preclinical efficacy by redirecting cytotoxic T lymphocytes toward AFP-expressing tumor cells. For instance, BsAbs engineered to recognize intracellular AFP peptides presented by HLA-A*02:01 (e.g., AFP158–166) and CD3 have shown potent tumor lysis both *in vitro* and *in vivo* HCC models [207]. While it’s a TCR-based bispecific (linking a TCR to an anti-CD3 scFv) rather than a direct antibody-antibody bispecific, it strongly supports the concept of AFP as a viable target for bispecific formats. These constructs exploit MHC-restricted presentation of tumor antigens to achieve high specificity, a notable innovation that extends the reach of BsAbs beyond surface-bound targets. While the use of AFP as a biomarker or therapeutic target in HCC immunotherapy faces notable limitations. Recent retrospective analyses reveal that the AFP positivity rate among patients with unresectable HCC (uHCC) has significantly declined over time, particularly with the increasing prevalence of non-viral HCC etiologies and earlier initiation of systemic therapies [221]. Specifically, AFP positivity dropped from 67.8% to 50.8% over the past 15 years, suggesting that a substantial proportion of uHCC patients no longer express AFP at levels sufficient for reliable detection or therapeutic targeting [221]. Thus, these limitations of AFP as a sole immunotherapeutic target in HCC highlight the need for combinatorial strategies to enhance both efficacy and patient coverage. Recent studies employing dual-targeted immunotherapies, such as CAR-T cells simultaneously directed against AFP and GPC3, provide a compelling rationale for applying similar principles to BsAbs development [208]. In preclinical models, AFP-GPC3 dual-targeted CAR-T cells demonstrated superior cytotoxicity, cytokine release, and tumor suppression compared to single-targeted AFP or GPC3 CAR-T cells, particularly in HCC tumors co-expressing both antigens [208]. This suggests that targeting AFP in conjunction with a second robust antigen like GPC3 may overcome the limitations posed by AFP’s variable expression and enhance immune engagement. Translating this concept to BsAbs platforms, AFP-based BsAbs could serve as supplementary components within dual-target constructs, thereby expanding therapeutic applicability and reinforcing antigen-specific cytotoxicity in heterogenous tumor populations.

## 3 Combination Therapies

BsAbs represent a rapidly evolving frontier in oncology, offering enhanced specificity and multi-modal functionality. While their monotherapy efficacy is promising, the future of BsAbs, particularly in the complex landscape of GI cancers, increasingly lies in sophisticated combination strategies and innovative technological advancements. The integration of BsAbs with conventional and emerging therapies holds significant promise for overcoming resistance and improving therapeutic outcomes in GI cancers. The integration of BsAbs with conventional and emerging therapies holds significant promise for overcoming resistance and improving therapeutic outcomes in GI cancers.

### 3.1 BsAbs with Chemotherapy

Chemotherapy remains a cornerstone of GI cancer treatment. BsAbs can synergize with chemotherapy by sensitizing cancer cells to cytotoxic agents, promoting immunogenic cell death, or by directly targeting tumor cells that have developed resistance to chemotherapy. The combination of the ERY974 with chemotherapy agents such as paclitaxel, cisplatin, and capecitabine has demonstrated synergistic antitumor efficacy in NCI-H446 and MKN45 xenograft tumor models [222]. While ERY974 monotherapy was limited by poor T-cell infiltration beyond the tumor-stromal boundary, co-administration with chemotherapy significantly enhanced T-cell penetration into tumor cores and increased intertumoral ERY974 distribution. In parallel, combination therapy using GPC3-targeted BsAbs and the chemotherapeutic agent Irinotecan has shown markedly enhanced therapeutic efficacy in HCC models [200]. The presence of Irinotecan supports this BsAbs significantly amplified tumor suppression and led to complete tumor growth arrest in multiple xenograft HCC models (HepG2, Hep3B, G1). Gemcitabine has been confirmed with the ability of enhancing the susceptibility of cancer cells to cytotoxic T lymphocyte (CTL)-mediated killing against chemo-resistant CCA [223], thus the combination therapy involving PD-L1 × CD3 BiTEs and gemcitabine has demonstrated enhanced cytotoxic efficacy against CCA by leveraging chemotherapy-induced immune modulation [224]. Gemcitabine not only sensitizes tumor cells but also upregulates PD-L1 expression, which can suppress T cell function. However, the use of PD-L1 × CD3 BiTEs-engineered to bind PD-L1 on CCA cells and CD3 on T cells, effectively reverses this immunosuppression, redirecting T cells to eliminate tumor cells. Two BiTE constructs, mBiTE and sBiTE, exhibited synergistic tumor killing in multiple CCA cell lines post-gemcitabine exposure, with the degree of cytotoxicity correlating with PD-L1 levels. These findings highlight the synergistic potential of BsAbs–chemotherapy regimens, particularly for enhancing tumor control in advanced GI cancer where monotherapies are insufficient.

Integrating BsAbs with conventional chemotherapy offers a multifaceted strategy to improve treatment outcomes in GI malignancies. However, while these combinations show promising preclinical results, several challenges remain, including optimizing dosing schedules to minimize overlapping toxicities, selecting patients with favorable tumor antigen profiles, and translating immunomodulatory synergy into durable clinical benefit. Future studies should aim to refine these strategies in biomarker-driven clinical trials to maximize the therapeutic index of BsAb-chemotherapy regimens in GI cancers.

### 3.2 BsAbs with ICIs

ICIs have revolutionized cancer treatment by enabling durable control of tumors that were previously deemed incurable or had reached advanced stages [225], the combination of BsAbs and ICIs is particularly compelling. BsAbs, especially T-cell engaging BsAbs, can overcome T-cell exclusion or anergy within the TME, thereby enabling ICIs to unleash a more robust and sustained anti-tumor immune response [79,226]. In preclinical models, a PD-L1 × CD3 BsAbs demonstrated limited efficacy as monotherapy but showed significantly enhanced tumor control when combined with regorafenib (REG), a tyrosine kinase inhibitor with immunomodulatory effects [227]. The REG-BsAbs combination synergistically promoted CD8^+^ T cell infiltration and cytotoxic activity in both MC38 and CT26 CRC models, shifting the TME toward a more proinflammatory (type 1) phenotype. Importantly, T cells from combination-treated mice exhibited stronger tumor cell reactivity than those treated with either agent alone. In preclinical humanized and syngeneic tumor models, TCB monotherapy significantly reduced tumor burden and induced robust infiltration and activation of intertumoral T cells [228]. Mechanistically, TCBs promote a pro-inflammatory microenvironment via CXCL10-CXCR3 signaling but also induce adaptive resistance through PD-1/PD-L1 upregulation. Combining TCBs with anti-PD-L1 antibodies enhances T cell infiltration and tumor control, highlighting their synergistic potential in overcoming resistance and improving outcomes in refractory GI cancers.

Despite promising synergistic effects, the combination of BsAbs and ICIs presents both opportunities and challenges. On one hand, this strategy offers a powerful dual mechanism: BsAbs actively recruit and engage T cells within otherwise immune-excluded tumors, while ICIs relieve inhibitory signaling, collectively driving deeper and more durable anti-tumor responses [226]. This is particularly relevant in GI cancers, where poor T cell infiltration and an immunosuppressive microenvironment often limit ICI efficacy alone [229]. However, the combination approach also raises concerns, such as enhanced immune activation leading to CRS or off-target toxicity, especially in solid tumors with low immunogenicity. Moreover, the optimal timing, dosing, and sequencing of BsAbs and ICIs remain to be standardized. Therefore, while the BsAbs-ICI combination represents a rational and potent therapeutic avenue, its clinical translation requires careful balancing of efficacy and safety, supported by biomarker-driven patient selection and further refinement through ongoing preclinical and clinical investigations.

## 4 Challenges

Despite the rapid advances in BsAbs–based immunotherapy for GI malignancies, several critical challenges remain that may limit their broader clinical translation and long-term efficacy. A more critical evaluation of these limitations is essential to guide rational BsAb design and optimize patient outcomes.

### 4.1 CRS

CRS represents the most characteristic toxicity associated with T-cell-engaging BsAbs. The immunopathological mechanisms of CRS were systematically elaborated: BsAb-mediated T-cell crosslinking triggers a cascading cytokine storm, with core mediators including IL-6, IFN-γ, and TNF-α, which further amplify immune activation through positive feedback loops [78,230,231]. Although these works provided a theoretical foundation for CRS management, its analysis was predominantly based on clinical experience in hematological malignancies, with limited discussion of the unique spatiotemporal characteristics of CRS in solid tumors, particularly GI cancers. In GI cancers, the clinical manifestations of CRS may be more complex, as intestinal epithelium is inherently highly sensitive to inflammation, and the dense stroma of the TME may restrict antibody distribution, resulting in sharply elevated local cytokine concentrations despite relatively normal systemic levels [20]. High tumor burden, heterogeneous antigen expression, and pre-existing inflammatory TMEs may exacerbate CRS risk [232,233,234].

#### 4.1.1 Current Management Frameworks of CRS and Their Limitations

Regarding CRS management, the consensus grading criteria published by the American Society for Transplantation and Cellular Therapy (ASTCT) in 2019 provided a standardized assessment framework [235]. This consensus stratifies CRS into grades 1–4, with clearly defined intervention thresholds for each grade. However, the application of this grading system to BsAb therapy in solid tumors presents significant limitations. Unlike CAR-T cell therapy, BsAb-induced CRS typically exhibits dose-dependent and reversible characteristics, yet the ASTCT grading does not adequately account for the complex relationship between CRS and therapeutic efficacy in solid tumors. For instance, in early-phase clinical trials of cibisatamab (CEA-TCB), severe grade 3–4 CRS events were observed [89], but the lack of a clear correlation between efficacy and toxicity rendered dose optimization exceptionally challenging.

#### 4.1.2 Lessons from First-Generation BsAbs: The Catumaxomab Experience

As the first approved BsAb (targeting EpCAM × CD3), catumaxomab’s clinical trajectory provides important cautionary lessons for BsAb development in GI cancers. A randomized controlled trial published by *Heiss* et al. in *The Lancet* demonstrated that intraperitoneal infusion of catumaxomab for malignant ascites significantly prolonged puncture-free survival (median 46 days vs. 11 days, *p* < 0.0001) [100]. However, this trial design harbored critical limitations: first, the study enrolled only patients with peritoneal metastasis, without evaluating therapeutic efficacy in systemic disease; second, the trial lacked a predefined CRS management protocol, resulting in a substantial proportion of patients being unable to complete the scheduled treatment course due to grade 3–4 toxicities (fever, nausea, abdominal pain). More importantly, catumaxomab’s Fc-mediated immune activation (through binding to FcγRI/FcγRIII), while conferring additional antitumor activity, significantly increased the risk of systemic inflammation. Clinical trial data revealed that 89% of patients developed CRS-related symptoms, including 10% with grade 4 severe events [236,237]. This safety profile ultimately led to catumaxomab’s market withdrawal in 2017, marking the failure of first-generation BsAbs in solid tumors. Critical analysis indicates that the trial by *Heiss* et al. overemphasized objective efficacy (ascites control) while neglecting patient-reported quality of life deterioration and long-term safety data—a trial design philosophy of “efficacy supremacy” that continues to resurface in subsequent BsAb development [100].

#### 4.1.3 Engineering Solutions and Their Clinical Limitations

In addressing CRS concerns, the design of CEA-TCB (cibisatamab) reported by *Bacac* et al. represented a significant engineering advancement [170,238]. This molecule employs an innovative 2:1 format (bivalent CEA binding, monovalent CD3 binding), which reduces systemic immune activation by lowering T-cell affinity (KD ~1 μM, substantially lower than ~1 nM for conventional BiTEs) while maintaining preferential binding to CEA-high-density tumor cells. Preclinical models demonstrated that this design expanded the therapeutic window approximately 5-fold, increasing the maximum tolerated dose from 0.5 mg/kg to 2.5 mg/kg. However, Phase I trial data for cibisatamab revealed the limitations of engineering optimization. Despite the promising preclinical profile of cibisatamab’s 2:1 structural design, clinical translation revealed persistent risks of severe CRS (including in patients with low CEA expression), a complex and poorly correlated relationship between toxicity and efficacy, and limited single-agent antitumor activity [89]. These findings offer critical insights for the next generation of T-cell-engaging BsAbs, highlighting the need for refined CD3 affinity modulation, rational combination strategies to enhance efficacy, and the development of predictive biomarkers to identify patients most likely to achieve a favorable risk–benefit profile.

Importantly, accumulating clinical evidence suggests that CRS severity does not necessarily correlate with antitumor efficacy, a phenomenon that complicates dose optimization and the determination of an optimal therapeutic window [239,240]. Current clinical management guidelines recommend premedication with corticosteroids (dexamethasone) and antihistamines, low-dose tocilizumab (8 mg/kg) for IL-6 pathway blockade, and intensive monitoring during the first infusion cycle [78,241,242,243]. Importantly, emerging data suggest that prophylactic tocilizumab addition to standard premedication regimens can significantly reduce CRS incidence without compromising antitumor efficacy, offering a promising approach to improve the therapeutic index of CD3-engaging BsAbs [244,245,246]. Although various mitigation strategies—including step-up dosing, affinity tuning of CD3-binding domains, and IL-6/IL-6R blockade—have been introduced to manage CRS, these approaches may inadvertently attenuate antitumor potency and remain incompletely standardized across clinical settings [247,248,249].

### 4.2 On-Target/Off-Tumor Effects

Beyond systemic toxicity, on-target/off-tumor effects constitute a major limitation of BsAbs in GI oncology. Many TAAs, including HER2, EGFR, and Claudin 18.2, are not strictly tumor-specific and are expressed at varying levels in normal gastrointestinal tissues [250,251]. BsAbs targeting such antigens—especially those engaging immune effector cells—may induce unintended cytotoxicity in normal tissues, even at low antigen density. This challenge is particularly pronounced for T-cell–redirecting BsAbs, where immune activation thresholds are low and tissue damage can occur despite modest antigen expression [19,252]. Consequently, achieving an optimal balance between tumor selectivity and immune potency remains a central challenge in BsAb design.

#### 4.2.1 The Solitomab Experience: A Cautionary Tale

The Phase I trial of solitomab, an EpCAM-targeting BiTE molecule, illustrated the formidable challenges facing BsAbs in GI cancers [99]. Enrolling patients with EpCAM-positive tumors—including gastric and colorectal cancers—the study found that most patients experienced DLTs below the target dose, including grade ≥ 3 liver enzyme elevations, diarrhea, and CRS. Ultimately, no MTD was established; the recommended Phase 2 dose was set at only 50% of the maximum evaluated dose. A critical finding was that high EpCAM expression in normal GI epithelium—particularly intestinal and biliary tissues—led to severe on-target/off-tumor toxicity [99]. The observed diarrhea and hepatotoxicity directly reflected damage to normal tissues. This outcome exposed a central paradox: given the potent cytotoxicity of T-cell engagers, even low-level expression of tumor-associated antigens in normal tissues can trigger irreversible damage. Notably, the trial required only tumor EpCAM positivity without an expression threshold, likely contributing to both suboptimal exposure in low-expression patients and heightened.

#### 4.2.2 Engineering Solutions: Masked BsAb (Probody) Technology

To mitigate on-target/off-tumor toxicity, masked BsAb (Probody) technology employs cleavable linkers at antigen-binding sites, enabling tumor-specific activation in MMP-rich microenvironments [253,254,255]. Preclinical evaluation of CI107 [255], an EGFR × CD3 Probody TCB, validates this approach: the masked molecule exhibited >500-fold reduction in antigen binding and >15,000-fold reduction in cytotoxic activity in healthy tissues, yet induced potent tumor regression upon protease-mediated activation in the TME. This tumor-specific activation translated into a substantially expanded therapeutic window, with the maximum tolerated dose in cynomolgus monkeys exceeding that of the unmasked TCB by more than 60-fold. Thus, by confining potent T-cell activation to the tumor site while sparing normal tissues, Probody technology addresses the fundamental safety-efficacy paradox of TCBs and expands their clinical potential for solid tumor indications. Nevertheless, the fundamental challenges persist. The absence of Probody-based TCEs in late-phase clinical trials underscores the gap between preclinical promise and clinical reality.

#### 4.2.3 The Antigen Sink Problem: Interference by Soluble Antigens

BsAb on-target/off-tumor toxicity arises not only from antigen expression in normal tissues but also from significant interference by soluble antigens (shed antigen) [256]. CEA, a prototypical secreted antigen, reaches serum concentrations of 100–1000 ng/mL in colorectal cancer patients—far exceeding the antigen saturation threshold required for therapeutic antibody efficacy. Serum CEA has been reported to achieve a sensitivity of 83.67% in the diagnosis of liver cancer [257]. Additionally, significantly higher serum CEA concentrations were observed in patients with HCC, suggesting a correlation with disease progression [258,259]. This “antigen sink” effect is particularly pronounced in GI cancers, as hepatic and peritoneal metastases continuously release substantial CEA into the circulation [260]. Although modern BsAb engineering approaches—such as 2:1 formats and high-affinity designs—attempt to address this issue, clinical data show limited success. This finding carries important implications for trial design: failure to stratify patients by serum CEA levels may lead to inappropriate enrollment of high-risk/low-benefit patients.

### 4.3 Tumor Heterogeneity

Tumor heterogeneity further undermines BsAb efficacy in GI malignancies. Both intertumoral and intratumoral heterogeneity in antigen expression can lead to incomplete tumor coverage and immune escape [261,262]. The progression of gastrointestinal stromal tumors (GISTs) under targeted therapy illustrates how tumor heterogeneity drives resistance. *Li* et al. found that among progressive GISTs, 34.8% exhibited multiple secondary mutation types, with heterogeneity observed both between and within tumor nodules [263]. This spatial diversity allows pre-existing resistant subclones to evade therapy and proliferate unchecked, leading to residual disease and relapse. The GIST paradigm underscores a fundamental challenge for BsAbs in gastrointestinal oncology: intratumoral heterogeneity enables immune escape, limiting response durability. Analysis of synchronous colorectal cancers revealed that distinct tumors within the same patient share remarkably few somatic variants (0.09%–0.36%). Crucially, concordance rates for driver genes such as KRAS, NRAS, BRAF, and PIK3CA were only 55.6%–66.7%, with discordant mutation subtypes frequently observed [264]. This genetic disparity means a BsAb effective against one lesion may be ineffective against another, creating an inherent resistance reservoir. Moreover, antigen expression can dynamically change under therapeutic pressure through epigenetic modulation or clonal selection, further limiting sustained BsAb engagement [265]. These observations highlight the necessity of rational antigen selection, dual- or multi-targeting strategies, and biomarker-driven patient stratification.

### 4.4 Acquired Resistance Mechanisms

In addition, intrinsic and acquired resistance mechanisms present significant obstacles to durable BsAb responses. Resistance may arise through antigen loss or downregulation, impaired immune synapse formation, or T-cell dysfunction and exhaustion following chronic activation [266,267,268]. In GI tumors, which are often characterized by dense stromal architecture and immunosuppressive cellular components—including regulatory T cells, myeloid-derived suppressor cells, and tumor-associated macrophages—BsAb-induced immune activation may be actively suppressed by the TME [269,270]. Furthermore, prolonged BsAb exposure may exacerbate T-cell exhaustion, reducing long-term cytotoxic capacity and immune memory formation [266,271]. T-cell exhaustion, characterized by progressive loss of effector function and upregulation of inhibitory receptors (PD-1, TIM-3, LAG-3), limits sustained antitumor activity [267]. Additionally, the immunosuppressive metabolite adenosine and regulatory cytokines (TGF-β, IL-10) within the TME can actively suppress BsAb-mediated T-cell activation [272,273], necessitating combination approaches with checkpoint inhibitors or TME-modulating agents [274,275].

### 4.5 Patient Stratification

A critical yet underexplored aspect of BsAb clinical development is biomarker-driven patient stratification. Predictive biomarkers for BsAb response remain inadequately defined, and their integration into trial design is essential for optimizing therapeutic outcomes. Antigen density thresholds represent one promising approach: preclinical studies have demonstrated that BsAb efficacy correlates with target antigen expression levels, suggesting that quantitative immunohistochemistry could identify patients most likely to benefit [86,276]. The relationship between target antigen density and BsAb efficacy has been rigorously elucidated through preclinical studies utilizing patient-derived organoid models. Using cibisatamab, a CEA × CD3 T-cell engager, *Teijeira* et al. demonstrated that colorectal cancer patient-derived organoids could be stratified into three distinct categories based on CEA expression: CEA-high (sensitive), CEA-low (resistant), and CEA-mixed populations that stably maintained both phenotypes [277]. Notably, organoids with heterogeneous CEA expression exhibited low sensitivity to cibisatamab, indicating that antigen-negative subclones sustain tumor growth under therapeutic pressure. Furthermore, sorted CEA-high and CEA-low cells from mixed populations demonstrated considerable plasticity in CEA expression, with antigen loss emerging as a mechanism of acquired resistance [86]. Complementary studies confirmed that cibisatamab-mediated tumor cell killing strictly depends on surface CEA levels, with a distinct expression threshold below which the low-affinity construct fails to induce T-cell cytotoxicity, whereas a higher-affinity CEACAM5-CD3 BsAb retained activity against low-expressing organoids [277]. These findings collectively establish antigen density as a critical determinant of BsAb efficacy and support the utility of quantitative immunohistochemistry for identifying patients most likely to benefit from CEA-targeted immunotherapies.

Collectively, these limitations underscore that while BsAbs represent a highly promising and versatile therapeutic modality, their successful application in GI oncology requires careful optimization of molecular design, dosing strategies, and patient selection. Integrating predictive biomarkers for toxicity risk, antigen heterogeneity, and immune competence—together with rational combination approaches involving ICIs, cytokine modulators, or stromal-targeting agents—will be critical to fully realizing the therapeutic potential of BsAbs in gastrointestinal cancers.

## 5 Future Directions

### 5.1 AI in Target Discovery and Biomarker Identification

Artificial intelligence (AI) is revolutionizing antibody discovery and engineering, offering unprecedented capabilities for BsAbs development. AI algorithms can analyze vast datasets of genomic, transcriptomic, and proteomic information from GI cancer patients to identify novel and highly specific tumor-associated antigens or resistance pathways [278,279,280]. By analyzing tumor RNA expression profiles, machine learning (ML) models achieved higher predictive accuracy—highlighting genes such as M6PR, IDO1, NRP1, and MAGEA3 as potential novel biomarkers in GI cancer [281]. These findings suggest that ML-guided biomarker discovery can refine patient selection and improve treatment precision in GI oncology. This accelerates the identification of optimal target pairs for BsAbs design, particularly in heterogeneous tumors. Leveraging next-generation sequencing (NGS) data, AI facilitates high-throughput, multi-modal characterization of TME components—enabling precise identification of prognostic and predictive biomarkers [282]. By integrating radiomics, transcriptomics, and immune profiling, AI algorithms enhance the resolution and interpretation of TME complexity, ultimately aiding in therapy stratification and the design of individualized treatment strategies. These advances support the transition toward precision oncology by optimizing chemotherapeutic and immunotherapeutic responses through refined TME assessment. Furthermore, artificial intelligence–driven models, such as the Genomic Mutation Signature (GMS), enable accurate prediction of survival outcomes in GI cancer patients receiving ICI therapy [283]. GMS outperformed traditional clinical markers in forecasting response and uncovered distinct immune landscapes, while also guiding targeted drug sensitivity—offering a precision oncology approach to optimize immunotherapy efficacy.

### 5.2 AI-Driven Structural Prediction and Antibody Optimization

Another notable application is the DiffDock model, a diffusion-based generative model for protein-ligand structure prediction [284]. When applied to antibody-antigen complex prediction, DiffDock achieved significantly higher accuracy than traditional docking methods, enabling more precise computational screening of BsAbs candidate structures before experimental validation. Such AI-driven structural prediction tools are particularly valuable for BsAbs design, where correct dual-target engagement depends on precise spatial orientation of binding domains. Stanford researchers have also developed machine learning-based methods for predicting antibody molecular changes that lead to improved drug properties [285,286]. Their approach enables rapid *in silico* screening of antibody variants, dramatically accelerating the lead optimization process that traditionally required extensive experimental iteration. For BsAbs development, such tools can simultaneously optimize binding affinity to both targets while maintaining favorable developability characteristics, potentially reducing the high failure rates associated with bispecific formats.

### 5.3 De Novo Antibody Design by Generative AI

A compelling example of AI in antibody development is the work by Absci Corporation, which employed generative AI models to *de novo* design functional antibodies with optimized developability profiles [287]. In their landmark study, AI-generated antibody sequences demonstrated comparable or superior binding affinity to naturally derived antibodies, while simultaneously optimizing key developability parameters such as solubility, stability, and low immunogenicity risk. This approach significantly reduced the traditional antibody discovery timeline from months to weeks, showcasing the transformative potential of AI-driven platforms in accelerating BsAbs development pipelines [288].

In summary, artificial intelligence offers transformative value in the development of BsAbs for GI cancers by enabling more precise target discovery, rational antibody design, and patient stratification. Through advanced integration of multi-omics datasets, including transcriptomics, proteomics, and radiomics, AI facilitates the identification of tumor-specific antigens and resistance pathways, guiding the construction of BsAbs with enhanced selectivity and efficacy. Furthermore, AI-driven predictive models, such as genomic mutation signatures, not only improve immunotherapy outcome forecasting but also uncover novel therapeutic targets. These capabilities underscore AI’s potential to accelerate BsAbs development and refine their clinical application within the framework of personalized GI cancer immunotherapy.

### 5.4 Emerging Frontiers: Nanomedicine, Synthetic Biology, and Personalized Neoantigen Targeting

Beyond AI, the convergence of precision nanomedicine and synthetic biology approaches holds substantial promise for developing next-generation intelligent BsAbs delivery systems. Integration of microenvironment-responsive nanocarriers with engineered BsAbs formats may represent a possible approach to overcome existing biological barriers, minimize systemic toxicities, and enable personalized, highly efficacious combination immunotherapies [289]. Moreover, patient-specific neoantigen targeting offers a compelling personalized therapeutic strategy for GI cancers by exploiting tumor-specific somatic mutations to generate aberrant peptides capable of eliciting robust immune responses with minimal off-target toxicity [290]. Recent advances in neoantigen identification and predictive algorithms have significantly improved the feasibility of designing individualized treatments, holding great promise for enhancing therapeutic efficacy and patient prognosis in GI cancer management. Although the application of such individualized targeting to BsAbs remains challenging, due to the complexity and urgency of personalized production, emerging progress in personalized vaccines and adoptive T cell therapies may ultimately enable the development of BsAbs formats that harness these uniquely tumor-specific antigens.

## 6 Conclusion

BsAbs have emerged as a transformative modality in the treatment of GI cancers, offering innovative strategies to overcome resistance to conventional therapies and ICIs. Recent advances have led to the successful development of BsAbs targeting tumor-associated antigens such as GPC3, CEA, EpCAM, and CLDN18.2, demonstrating potent antitumor efficacy through T-cell redirection, immune modulation, and TME reprogramming. Additionally, BsAbs have shown synergistic potential when combined with chemotherapy, radiotherapy, immune checkpoint blockade, or novel co-stimulatory agents, further expanding their therapeutic scope across diverse GI malignancies, including pancreatic, colorectal, gastric, cholangiocarcinoma, and hepatocellular carcinomas.

Looking ahead, the next phase of BsAbs development will require integrating AI tools for rational target selection, structure optimization, and patient stratification to enhance precision and efficacy. Future research should also explore tri- and multispecific formats, conditionally active BsAbs, and combinatorial regimens that can better address tumor heterogeneity and immune evasion. Furthermore, incorporating insights from spatial multiomics, radiogenomics, and TME profiling will refine therapeutic decision-making and patient selection. Furthermore, five critical issues merit priority attention in future research: (1) establishing standardized biomarker panels for patient selection, including validated antigen density thresholds and immune infiltration metrics; (2) developing next-generation BsAbs formats with enhanced tumor selectivity and reduced on-target/off-tumor toxicity; (3) optimizing combination strategies through biomarker-driven clinical trial designs; (4) implementing systematic CRS risk prediction algorithms to improve safety profiles; and (5) integrating AI-guided approaches throughout the drug development continuum, from target discovery to clinical decision support.

To accelerate translation into clinical benefit, large-scale, multicenter trials and interdisciplinary collaborations between immunologists, oncologists, structural biologists, and computational scientists are essential. Only through such collective efforts can we fully harness the therapeutic potential of BsAbs and shape a more effective and individualized treatment paradigm for patients with GI cancers.

## Data Availability

Not applicable.
